# Osteopontin in Alzheimer's Disease: A Double‐Edged Sword in Neurodegeneration and Neuroprotection—A Systematic Review

**DOI:** 10.1111/cns.70269

**Published:** 2025-02-17

**Authors:** Zahra Azizan, Maryam Bazrgar, Narges Bazgir, Sadra Habibi Moini, Sara Ghaseminejad‐Kermani, Kamran Safa, Azam Eshaghian‐dorcheh, Mohammad Hossein Harirchian

**Affiliations:** ^1^ Department of Neurology, School of Medicine, Iranian Center of Neurological Research, Neuroscience Institute, Imam Khomeini Hospital Tehran University of Medical Sciences Tehran Iran; ^2^ Neuroscience Research Center Shahid Beheshti University of Medical Sciences Tehran Iran; ^3^ Hearing Disorders Research Center, Loghman Hakim Hospital Shahid Beheshti University of Medical Science Tehran Iran; ^4^ Emergency Medicine Department Shahid Beheshti University of Medical Science Tehran Iran; ^5^ Kashani Hospital Isfahan University of Medical Sciences Isfahan Iran

**Keywords:** Alzheimer's disease, microglia, neuroinflammation, osteopontin, secreted phosphoprotein 1, synaptic pruning

## Abstract

**Background:**

Osteopontin (OPN) has emerged as a pivotal molecule in Alzheimer's disease (AD), with studies indicating its potential to act as both a neuroprotective agent and a contributor to neurodegeneration. This systematic review aims to elucidate the roles of OPN in AD pathogenesis through inflammatory pathways.

**Methods:**

We conducted a comprehensive analysis of current literature on OPN's involvement in AD, focusing on its signaling pathways, cellular interactions, and regulatory mechanisms. We searched PubMed, EMBASE, and Scopus databases by the keyword of Alzheimer's Disease and Osteopontin. Our date search was in 1990 until July 1, 2024 with no language limitation.

**Results:**

In a review of 758 studies, a total of 15 reports met the eligibility criteria and were included. Among the findings, four studies provided evidence supporting the protective mechanism of OPN within the context of AD. Eleven studies explain the inflammatory role of OPN. OPN has been shown to play a role in synaptic pruning, microglial activation, and the inflammatory processes associated with AD. Additionally, OPN is implicated in facilitating cellular communication and serves as a chemotactic molecule. It is suggested that the protective effects of OPN are predominantly mediated by the c fragment of the protein and are most prominent in the early stages of AD progression.

**Conclusion:**

OPN in AD has dual effects—protecting neurons and contributing to their degeneration. Future research should enhance its protective mechanisms, target specific signaling pathways, and develop therapies to slow AD progression.

## Graphical abstract

Osteopontin (OPN) is secreted by activated microglia, astrocytes, and infiltrating immune cells. OPN exhibits dual roles in Alzheimer's disease (AD), acting as a double‐edged sword with both protective and detrimental effects. OPN contributes to AD progression through mechanisms leading to synaptic pruning, microglial activation, innate immune system activation, and chemotactic signaling. Conversely, OPN under certain conditions plays a protective role by maintaining synaptic integrity, facilitating vascular amyloid‐beta (Aβ) clearance, and activating microglia to enhance Aβ clearance.

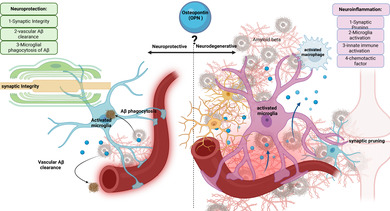

## 1 Introduction

Alzheimer's disease (AD), the most prevalent form of dementia worldwide [1], is a progressive neurodegenerative disorder characterized by the aggregation of amyloid‐beta (Aβ) plaques and hyperphosphorylated tau protein in neurofibrillary tangles (NFTs) within the brain parenchyma [2]. Despite significant research efforts, the intricate mechanisms underlying AD pathogenesis remain largely unresolved. A deeper understanding of these pathways is crucial for developing effective therapeutic strategies, as current interventions primarily focus on managing symptoms rather than halting disease progression [3].

Recent investigations have implicated chronic neuroinflammation as a key contributor to Aβ accumulation and subsequent neurodegeneration in AD [4]. Microglia, the brain's resident immune cells, play a pivotal role in this process. Upon encountering Aβ plaques, microglia become activated and adopt a proinflammatory phenotype, releasing a cascade of inflammatory mediators such as cytokines, chemokines, and reactive oxygen species (ROS). These mediators can further exacerbate neuroinflammation and neuronal dysfunction, perpetuating the pathological cycle [5].

Osteopontin (OPN), encoded by the secreted phosphoprotein‐1 (SPP1) gene, has emerged as an intriguing player in this neuroinflammatory milieu [6]. OPN is a multifunctional glycoprotein with diverse roles in cell adhesion, migration, and various signaling pathways. Notably, SPP1 expression has been observed to be upregulated in the brains of AD patients and animal models, suggesting its potential involvement in disease progression [7].

While in vitro and in vivo studies have demonstrated OPN's ability to activate inflammatory pathways like NF‐κB, MAPK, and ERK, contributing to neuroinflammation, other studies suggest a more complex role [8]. OPN might also possess neuroprotective properties, potentially aiding in Aβ clearance or dampening excessive inflammation. This duality necessitates a comprehensive analysis to elucidate the multifaceted contribution of OPN to the neuroinflammatory cascade in AD [9].

The absence of a systematic approach to understanding the dual role of OPN in disease progression has prompted us to address this critical knowledge gap. This review aimed to systematically evaluate the existing literature on the seemingly contradictory roles of OPN in AD. By critically analyzing both in vivo and in vitro studies, we seek to elucidate the molecular mechanisms through which OPN influences inflammatory pathways in AD, with a focus on identifying key signaling cascades and their downstream effects. Through this comprehensive review, we aimed to provide a clearer understanding of OPN's complex contributions to disease pathogenesis, ultimately guiding future research efforts.

## 2 Method

### 2.1 Protocol Registration

To ensure transparency and minimize reporting bias, the protocol for this systematic review was prospectively registered with the International Prospective Register of Systematic Reviews (PROSPERO) (https://www.crd.york.ac.uk/prospero/), which was assigned the ID CRD42024545946.

### 2.2 Search and Study Selection

The main question for this systematic review is “In animal models and in vitro models of AD, how does the modulation of SPP1/OPN compare to control groups, affect inflammatory pathways and disease progression?”

We conducted a systematic search across three databases, PubMed, Scopus, and Embase, extracting articles without language limitations; the search date was from 1990 to July 1, 2024. Additionally, we manually searched the gray literature, including conference papers and thesis. The search query employed the “Alzheimer's Disease AND (‘Osteopontin’ OR ‘Secreted phosphoprotein 1’).” A detailed search strategy is available in Data S1. The Population, Intervention, Comparator, Outcome (PICO) framework of our study encompassed the following:

- P: in animal models and in vitro models of AD
- I: modulation of SPP1/OPN
- C: control groups
- O: inflammatory pathways and disease progression

Duplicate articles were removed using reference management software (Mendeley). Initial screening was performed by researchers based on titles, followed by an abstract. Then, the selection process was carried out according to the eligibility criteria, independently by two researchers. In cases of disparity, a third expert researcher made the final decision on article selection.

### 2.3 Eligibility Criteria

Exclusion criteria involved any articles related to dementia types other than AD (e.g., HIV‐induced dementia, vascular dementia, Lewy bodies, or frontotemporal dementia), non‐dementia‐related diseases, review articles, bioinformatics studies, randomized controlled trials, editorials, commentaries, or diagnostic or biomarker studies. Original articles or conference papers specifically discussing the role of SPP1/OPN in AD were included. Detailed eligibility criteria are described in Table 1.

**TABLE 1:** Eligibility criteria.

Elements	Inclusion	Exclusion
P (population)	Animal: any types of animal model of Alzheimer's disease (i.e., rat or mice) in in vivo studies with all species, sex and age, and genetically modified included In vitro studies: cell cultures of microglia, neurones, macrophages, astrocytes, and or immortalized cell lines expressing included Alzheimer's disease phenotypes	Human studies, non Alzheimer's disease animal model, or other type of dementia model studies, ex vivo, in silico models
I (Intervention)	Animal: SPP1/OPN modulation (up or downregulation) or use of drugs affecting SPP1/OPN expression, genetic modulation (knockdown or overexpression of SPP1/OPN) In vitro: SPP1 inhibitors (i.e., siRNA or antibodies) or other compound affecting SPP1/OPN	Studies where SPP1 is not the part of the intervention
C (comparison)	Animal: Wild‐type animals In vitro: cells without genetic or experimental modification of SPP1/OPN	Comparison groups with intervention
O (outcome)	Studies reporting on inflammatory markers (e.g., cytokine levels, microglial activation) and/or Alzheimer's disease progression indicators (e.g., behavioral assessments, plaque formation, neurodegeneration, cellular changes)	Studies not reporting relevant inflammatory markers or Alzheimer's disease progression indicators. Studies with outcomes not related to the inflammatory pathways or Alzheimer's disease progression
Others	All primary experimental research articles without time or language limitation	Review, editorial, commentaries, and letters will be excluded. Any type of study except experimental such as cohort, observational, and case–control

### 2.4 Data Extraction

Included articles were analyzed for publication date, study design, authors, and the role of SPP1 through various pathways. Two researchers conducted data extraction independently, and the accuracy of the retrieved information was verified by a third expert researcher with a third expert researcher validating the accuracy of the information retrieved. Table 2 shows the details of data to be extracted.

**TABLE 2:** Extracted data.

Elements	Extracted data from in vivo studies	Extracted data from in vitro studies
Population	Species, sex, age, type of Alzheimer's model, mice or rat, method of Alzheimer's disease induction (e.g., genetic modification, chemical induction)	Cell type (e.g., primary neurons, microglia, astrocytes), source of cells (e.g., transgenic animals, human‐derived cell lines), culture conditions
Intervention	Method of SPP1 modulation (e.g., genetic overexpression, knockdown, pharmacological agents)	Method of SPP1 modulation (e.g., genetic overexpression, knockdown, pharmacological agents)
Primary outcome	Signaling pathway, markers of inflammation signaling pathways measured (e.g., cytokine levels such as TNF)	Cellular changes in vitro (e.g., cell viability, protein aggregation)
Secondary outcomes	Histopathological changes (e.g., amyloid plaque load, tau pathology, neuronal loss), behavioral assessments (e.g., Morris water maze, Y‐maze, open field test), methods used for measuring inflammatory markers (e.g., ELISA, Western blot, immunohistochemistry), the type of cells producing SPP1/OPN, the triggers that cause SPP1/OPN production, any noted pattern or key findings	Histopathological changes (e.g., amyloid plaque load, tau pathology, neuronal loss), methods used for measuring inflammatory markers (e.g., ELISA, Western blot, immunohistochemistry), the type of cells producing SPP1/OPN, the triggers that cause SPP1/OPN production, any noted pattern or key findings
Others	Year, published date, published journal, authors, study design	Year, published date, published journal, authors, study design

### 2.5 Risk of Bias (RoB) Assessment

The RoB for each study was independently assessed by two reviewers who worked separately. This independent assessment helped ensure objectivity and reduce personal bias in the evaluation process. Discrepancies between the two reviewers were resolved by a third reviewer, who acted as a mediator in cases where the two initial reviewers disagreed on the RoB assessment. This three‐reviewer approach ensured the accuracy and consistency of the bias evaluations.

For animal studies, we employed SYRCLE's RoB tool, which evaluates key domains including selection bias, performance bias, detection bias, and reporting bias. In vitro studies were assessed based on the clarity of experimental protocols, the use of appropriate control groups, and the reporting of outcome measures.

## 3 Result

### 3.1 Overview

**FIGURE 1:**
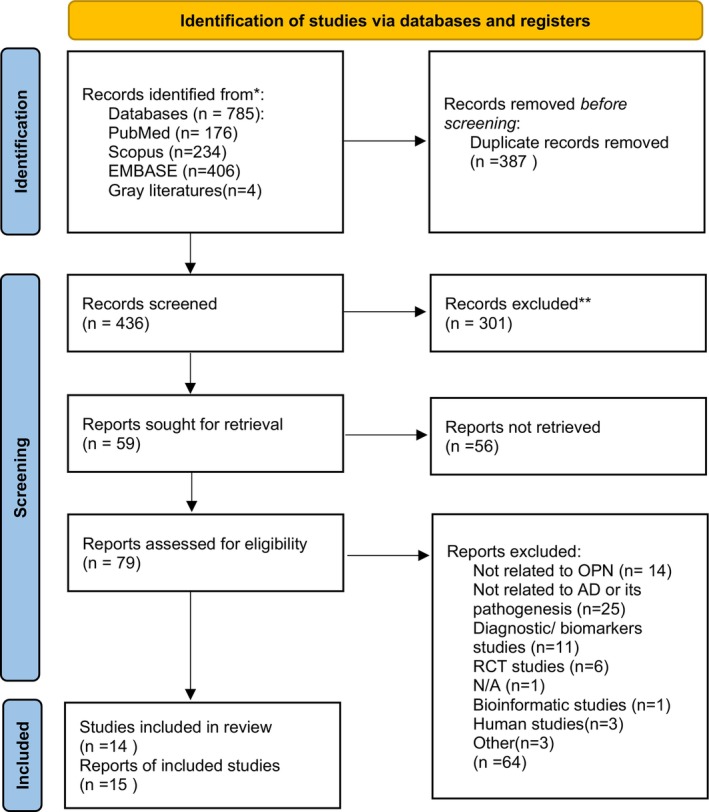
PRISMA flowchart. From: Page et al. [52]. *Consider, if feasible to do so, reporting the number of records identified from each database or register searched (rather than the total number across all databases/registers). **If automation tools were used, indicate how many records were excluded by a human and how many were excluded by automation tools.

Out of the initial set of 785 studies identified in the database search, 436 studies were retained after eliminating duplicates. Subsequent screening resulted in the selection of 79 studies, with 357 studies being excluded based on title and summary evaluation. Following assessment against eligibility criteria, a total of 15 studies met the inclusion criteria as illustrated in Figure 1 of the PRISMA flow chart. Among these, seven studies were conducted in both in vivo and in vitro settings, whereas one study, defined as a thesis, encompassed both in vivo and in vitro components. The remaining studies exclusively focused on in vivo experiments. A detailed summary of these studies is provided in Tables 3 and 4.

**TABLE 3:** Studies' description: neurodegenerative role of OPN.

Study details/reference	In vivo/in vitro	Type of study	Animal/human (*n* = number)	Tissue	SPP1 modulation	Main outcomes	OPN producer	Trigger(s)
Yang et al. (2019) [18]	In vivo: B6.APP/PS 1 mice	Experimental	4–6 mice/group	Brain (prefrontal and motor cortex)	Chronic consumption of a western diet	OPN + IBA1 cells, activated neutrophils, exhibited an elevation in the cerebral regions of mice subjected to a western diet through a CCR2‐dependent mechanism SPP1 has the potential to augment the inflammatory response in microglia, resulting in heightened activation of TREM1 Spp1 expression was not detected in microglia that expressed high levels of Aif1^a^ Obesity activates the Itgal, Trem1, and Spp1 pathways within brain myeloid cells	CD11b + CD45hi cells (activated microglia and infiltrating peripheral myeloid cells) and OPN + IBA1−(activated notrophils) cells	Western diet
Sala Frigerio et al. (2019) [11]	In vivo: Male APP NL‐G‐F—	Experimental	5–7/group	Cortex and hippocampal	N/A	The genes responsible for tissue repair, including Spp1 and Dkk2, selectively expressed by cells located at ARMs belonging to State 3 branch ARM is characterized by the upregulation of genes associated with inflammatory processes (Cst7, Clec7a, and Itgax), major histocompatibility complex (MHC) class II antigen presentation (Cd74, H2‐Ab1, H2‐Aa, Ctsb, and Ctsd), and tissue regeneration (Spp1, Gpnmb, and Dkk2)	ARM	Amyloid beta
De Schepper et al. (2023) [15]	In vivo: App NL‐F In vitro: Primary mouse microglia	Experimental case–control	Animal: 20/group Human: AD (*n* = 6), control (*n* = 6)	Hippocampal CA1 stratum	SPP1 knockdown	OPN, acting through its integrin receptor on activated microglia, facilitates synaptic engulfment SPP1 facilitates perivascular‐microglial cross talk through autocrine TGF‐β signaling Additional predicted Spp1‐specific pathways in the microglia of AppNL‐F mice include Calr^b^ Early vascular Aβ deposition occurs coinciding with SPP1 activation in the perivascular space in the AppNL‐F mouse model before plaque formation SPP1 is primarily expressed by CD206 + CD163 + Pf4 + PVMs and Pdgfra+/CD140 + PVFs in the hippocampus during the onset of microglia‐mediated synapse engulfment Perivascular SPP1 expression is observed in the CA1 region of the hippocampus in AD patient brains	PVM and PVF	Oligomer amyloid beta
Hong S. (2022) [30]	In vivo: transgenic	Experimental case–control (conference paper)	ADtg	Brain	SPP1 knockdown	SPP1 mRNA and protein levels are upregulated during the period of complement deposition, coinciding with the increased vulnerability of synapses to microglial engulfment In SPP1‐knockout mice, Aβ oligomers fail to induce synapse loss, unlike in wild‐type controls	PVM and PVF	
Wirths et al. (2010) [22]	In vivo: APP/PS1 K1 mice	Experimental/	ADtg = 23 Control = 22	Brain	Expression patten of OPN in AD	Notably, TGF‐β1 and OPN were the only cytokines to exhibit significant upregulation at 6 months of age in APP/PS1KI mice Double staining with Aβ and OPN antibodies revealed clear OPN immunoreactivity within the neuritic component of extracellular plaques	N/A	Amyloid beta
J‐H Park et al. (2017) [10]	In vivo: 3xTg‐AD mice	Experimental	N/A	Plasma	High‐fat diet	HFD‐fed 3xTg‐AD mice exhibited significantly elevated plasma levels of inflammatory and metabolic markers, including L‐selectin, sTNF RII, IGFBP‐2, MMP‐3, resistin, and osteopontin, compared to ND‐fed counterparts	N/A	HFD
Qiu et al. (2023) [17]	In vivo: B6.Cg‐Tg In vitro: Microglia isolated from 9‐mo‐old OPN‐KO.5XFAD mice	Experimental case–control	Animal: Human: cognitively normal (*n* = 11) Mild cognitive impairment (MCI, *n* = 10) AD patients (*n* = 11)	Brain	In vivo: OPN knockdown, administration of anti‐OPN mAb In vitro: OPN knockdown added rmOPN in in vitro microglial cultures	OPN production differentiates CD11c+ microglia into pathogenic (CD11c+OPN+) and protective (CD11c+OPN−) subsets Interaction between OPN and its αVβ3 integrin receptor induces TNF‐α production and inflammasome activation, converting homeostatic microglia into a proinflammatory state that impairs efficient Aβ phagocytosis OPN promotes a proinflammatory response in CD11c + microglia through interaction with the αVβ3 integrin receptor and may inhibit Aβ plaque compaction by suppressing the TREM2/TAM‐lysosomal phagocytic pathway AB➔activated microglia➔ ↑ OPN ➔ OPN + avb3 integrin➔ ↑ caspase‐1 activity and IL‐1b (activation of microglial inflammasome) Deletion of OPN significantly improved cognitive functions, reduced microglial production of the TNF‐α levels, decreased Aβ plaque area by 40%–60%, reduced dystrophic neurites per plaque by 50%. OPN deficiency increased the expression of TREM2, Axl, and Mer in CD11c + microglia, enhancing lysosomal activation as evidenced by higher CD68 and cathepsin B levels. This activation likely facilitated Aβ degradation	CD11+ microglia	Amyloid beta
Lai et al. (2021) [13]	In vivo: F344TgAD rats	Experimental	6 rat/per group	Cingulate and somatosensory cortex	The modulation of SPP1 levels was a result of the transient hypertension induced by L‐NAME administration	OPN and ROCKs have been implicated in the pathogenesis of hypertension‐related vascular remodeling OPN expression was significantly lower in hypertensive transgenic AD rats compared to non‐transgenic AD rats Transient hypertension, but not AD, was associated with a significant decrease in OPN levels, highlighting its potential role in vascular alterations Key downstream effectors of OPN signaling include the Rho–associated protein kinases (ROCKs), ROCK1, and ROCK2	Smooth muscle and endothelial cells	HTN
Chuang, Yi (2018) [21]	In vivo: APP/PS1 mouse In vitro: Brain cortices and hippocampi/cortical neuron of 11 days in vitro (DIV)	Experimental (thesis)		Cerebral cortices	Two doses of murine recombinant IL‐33 (580506, BioLegend) was intraperitoneally (i.p.) injected to 12 month‐old female mice (200 ng each day per mouse) in 2 consecutive days	OPN has been shown to activate the Akt and p38 MAPK pathways in cortical neurons. Chronic brain inflammation in transgenic APP/PS1 mice may drive astrocyte activation, leading to alterations in the OPN/CD44 signaling pathway IL‐33 modulates the OPN/CD44 signaling pathway in APP/PS1 mice, a mechanism potentially altered by chronic brain inflammation SPP1 expression is significantly upregulated in the cerebral cortex of aged APP/PS1 mice and is also detected in the thalamic reticular nucleus OPN is also induced in microglia with glial‐like morphology OPN‐expressing microglia are closely associated with amyloid‐β plaques OPN is prominently expressed in microglia involved in phagocytosis in APP/PS1 mice, with the majority being in its secreted form (sOPN)	GABAergic neurons Activated microglia	Amyloid beta
Gharpure et al. (2024) [28]	In vivo: APP/PS1 mouse	Experimental	APP/PS1 mouse	Cortex	SPP1 regulation	The inflammatory response is modulated by SPP1/OPN via macrophage chemotaxis and has been investigated in the context of DAM in AD Alzheimer's mice exhibit increased expression of SPP1/OPN and CSF‐1^c^ compared to wild‐type mice	Microglia	Amyloid beta
Wang et al. (2020) [27]	In vivo: 5XFAD mice In vitro: bone marrow macrophage	Experimental	5XFAD mice	Brain	Systemic administration of AL002c (TREM2 agonist)	AD mice showed elevated expression of SPP1/OPN and CSF‐1^c^ AL002c was linked to the downregulation of Spp1/OPN expression within Iba1+ cells compared to control and increased the ratio of Aβ42+CD68+ microglial phagosomes to total Aβ42, indicating microglial activation and enhanced phagocytosis of Aβ TREM2➔reduce inflammatory markers SPP1/OPN➔ameliorates Aβ plaque	Iba + cells (microglia)	Amyloid beta

Abbreviations: AD, Alzheimer's disease; ADAM17, a disintegrin and metalloproteinase 17; Akt, protein kinase B (PKB); ARM, activated microglia; Aβ, amyloid beta; CAA, cerebral amyloid angiopathy; CC, cingulate cortex; CCR2, C‐C chemokine receptor type 2; DAM, disease‐associated microglia; EC, entorhinal cortex; HC, hippocampus; HFD, high‐fat diet; HTN, hypertension; Itga, integrin alpha; KO, knock down; L‐NAME, Nω‐Nitro‐L‐arginine methyl ester; MAPK, mitogen‐activated protein kinase; MMP, matrix metalloproteinase; OPN, osteopontin; PVF, perivascular fibroblasts; PVM, perivascular macrophage; SPP1, secreted phosphoprotein 1; TGF‐1, transforming growth factor 1; TGF‐β, transforming growth factor beta; TNF‐α, tumor necrosis factor alpha; TREM/TAM, triggering receptor; expressed on myeloid cells/Tyro3, Axl, and MerTK; TREM, triggering receptor expressed on myeloid cells; WT, wild type.

^a^ Aif1 the gene for IBA1, a marker for microglia and monocytes/macrophages.

^b^ Calr encodes calreticulin, a multifunctional chaperone protein recognized as an “eat‐me” signal.

^c^ CSF‐1 playing a critical role in synaptic pruning.

**TABLE 4:** Studies' description: neuroprotective role of OPN.

Study details/reference	In vivo/in vitro	Type of study	Animal/human (*n* = number)	Tissue	SPP1 modulation	Main outcomes	OPN producer	Trigger(s)
Rentsendorj et al. (2018) [31]	In vivo: (ADtg)B6Cg‐Tg In vitro: MφBM from WT or OPN KO mice	Experimental case–control	Animal: 4/proup Human: 2/group	Animal: HC, CC, EC Human: postmortem brain from frontal	In vivo: OPN KO In vitro: OPN siRNA	MФBM phagocytose pathogenic Aβ fibrils depend on OPN expression Treatment with GA enhanced MФBM phagocytosis activity of Aβ through a gain‐of‐function mechanism mediated by elevated OPN expression Increased expression of OPN and subsequent inhibition of NO and iNOS may serve as a potential mechanism through which macrophages mitigate neuroinflammation in AD C‐terminal OPN fragments created by MMP‐9 releasing by macrophages OPN promotes an anti‐inflammatory macrophage phenotype	Mo/MФ (selectively Iba1 + CD45 high)	Amyloid‐beta fibrils
Li et al. (2020) [32]	In vivo: B6Cg‐Tg In vitro: bone marrow‐derived macrophages	Experimental	6/group	Hippocampal area ENT (entorhinal cortex)	Upregulates OPN expression by GA(Glatiramer acetate)	Elevated Mo/M8–mediated OPN/SPP1 expression in the brain has been associated with the maintenance of synaptic integrity and cognitive function Synaptogenesis was heightened in the presence of M8BM overexpressing OPN in the brains of immunized ADtg mice	Macrophages	Amyloid‐beta oligomers
Quan et al. (2021) [34]	In vivo: APP/PS1‐transgenic mice In vitro: human primary brain vascular pericytes (HBPC)	Experimental	4/group	Cortex and hippocampus	Haploin sufficiency of MyD88	Decreased MyD88 expression in microglia was found to enhance brain vasculature in transgenic mice, correlating with upregulated OPN and IGF‐1 gene transcription in microglia Microglia with reduced MyD88 expression also upregulated LRP1 protein levels in cerebral capillaries, further facilitating Aβ clearance MyD88 haploinsufficiency significantly suppressed proinflammatory gene transcripts, including TNF‐α and IL‐1β	CD11b + brain cells: microglia and infiltrating macrophages	Myd88 deficiency
Zhang et al. (2022) [12]	In vivo: male APP/PS1 mice	Experimental	20 mice/per group	Hippocampus	Running exercise	Exercise promotes microglial phagocytosis, potentially by increasing SPP1 expression, which enhances activation of the TREM2/SYK signaling pathway SPP1 was significantly increased in running AD mice, while ADAM10, lower in sedentary AD mice compared to wild‐type, showed improved expression with running, hinting at alternative mechanisms, and affecting TREM2 hydrolysis	DAM	Exercise

Abbreviations: AD, Alzheimer's disease; ADAM17, a disintegrin and metalloproteinase 17; Aβ, amyloid beta; CC, cingulate cortex; DAM, disease‐associated microglia; DG, dentate gyrus; EC, entorhinal cortex; fAβ42, fibrillar Aβ42; GA‐Mφ, glatiramer acetate–treated macrophages; HC, hippocampus; IGF‐1, insulin–like growth factor 1; iNOS, inducible nitric oxide synthase; Itga, integrin alpha; KO, knock down; MMP, matrix metalloproteinase; MyD88, myeloid differentiation primary response 88; MФBM, bone marrow–derived macrophages; NO, nitric oxide; oAβ42, oligomeric Aβ42; OPN, osteopontin; OS, stratum oriens; PVFs, perivascular fibroblasts; PVM, perivascular macrophage; SLM, stratum lacunosum moleculare; SPP1, secreted phosphoprotein 1; SR, stratum radiatum; SYK, spleen tyrosine kinase; TGF‐β, transforming growth factor beta; TGF‐β, transforming growth factor β; TREM, triggering receptor expressed on myeloid cells; WT, wild type.

### 3.2 OPN Producers and Triggers

Risk factors for AD, such as a high‐fat diet [10], female gender, the APOE gene [11], sedentary lifestyle [12], hypertension [13], and aging [11], are known to induce the production of OPN. This leads to a transformation of the hemostatic phenotype of microglia into an activated state, an increase in macrophage infiltration, and extracellular matrix remodeling. Research has shown a correlation between OPN and immune responses within neural cellular plaques, indicating the triggering role of amyloid beta in OPN production [14].

Although most studies link high blood pressure with increased OPN expression, a recent study found a reduction in OPN levels following an induction of transient hypertension in an AD model. The study suggested that the rho‐associated protein kinases (ROCKs) may be a key target influenced by OPN. Activation of this signaling pathway could potentially contribute to brain tissue damage and vascular alterations in AD models and aging mice [13]. Research conducted by De Schepper et al. [15] has shown that OPN is primarily secreted by perivascular macrophages (PVMs) and perivascular fibroblasts (PVFs). Other studies suggest that OPN is also secreted by infiltrating macrophages, activated microglia, astrocytes, and vascular endothelial cells. This highlights the potential role of OPN as a cell communicator in pathological conditions, suggesting that PVM and PVF may serve as initiators of OPN signaling and inflammatory cascades [16].

### 3.3 Neurodegenerative

#### 3.3.1 Overview

A review of 11 studies has revealed a consistent involvement of OPN in neuroinflammation processes. Four of these studies utilized both in vivo and in vitro models, whereas the remaining seven focused solely on in vivo investigations. To bridge the gap between animal models and human pathophysiology, five studies incorporated human cohorts. The overall evidence strongly suggests a proinflammatory function for OPN. Noteworthy, the dataset comprised one thesis and two conference abstracts, with one abstract later being published in a peer‐reviewed journal (Table 3).

#### 3.3.2 OPN Induces Production of Inflammatory Cytokines

In 5XFAD transgenic mice lacking OPN (OPNKO), there is a notable reduction in the production of the proinflammatory cytokine TNF‐α, Aβ plaque levels, and diminished presence of dystrophic neuritis in the brain. Conversely, administration of recombinant OPN leads to elevated TNF‐α production and increased inflammation. These observations highlight the critical role of OPN/αVβ3 signaling in promoting TNF‐α production specifically in CD11c + microglia, thereby fostering neuroinflammatory processes [17]. Furthermore, De Schepper et al. [15] have identified alterations in TGF‐β1 levels in mice deficient in the SPP1 gene, indicating OPN's involvement in modulating perivascular‐microglia communication through the TGF‐β autocrine pathway.

Although the precise mechanisms governing the interaction between OPN and TGF‐β remain incompletely elucidated, a synergistic relationship is proposed wherein TGF‐β and OPN signaling collectively contribute to neuroinflammation. The study also implicates ADAM17 and calreticulin as potential ligands for OPN in PVM and PVF, suggesting their involvement in the intricate interplay between OPN, ADAM17, and calreticulin that ultimately culminates in amplified inflammatory responses [15].

#### 3.3.3 OPN Induces Microglial‐Mediated Synaptic Pruning

Upon exposure to inflammatory stimuli, OPN is released and binds to integrin receptors. This binding event triggers intracellular signaling cascades that result in the upregulation of inflammatory cytokines, such as TNF‐α, and phagocytic markers (C1qa, Grn, and Ctsb) in microglia associated with Aβ oligomers. The presence of C1q, secreted by microglia, promotes the activation of the complement component C3 to C3b on synaptic surfaces. C3b then binds to C3bR microglial receptors, leading to synaptic engulfment [15, 17].

The absence of OPN leads to diminished phagocytic activity in microglia, despite their inherent phagocytic potential. These findings underscore the essential role of OPN as an extracellular signaling molecule that primes microglia for phagocytosis and synaptic engulfment [15]. The expression levels of genes encoding the C1q complex are increased in CD11b + CD45hi/low cells, representing activated microglia and peripherally derived myeloid cell [15, 18]. Additionally, elevated levels of Itga1, TREM1, and SPP1 indicate the involvement of infiltrated activated myeloid cells in inflammatory responses and suggest potential cross talk between OPN and innate immune cells in synaptic pruning through complement cascade activation [18].

#### 3.3.4 OPN Activates Akt and p38 MAPK Signaling

Akt, a serine/threonine kinase, plays a crucial role in regulating neuronal survival, insulin signaling, and synaptic plasticity [19]. In contrast, p38 MAPK is a significant mediator of proinflammatory responses, apoptosis, and tau protein hyperphosphorylation [20]. The delicate balance between these kinases is essential for maintaining neuronal health.

OPN, a multifunctional cytokine, can selectively modulate these pathways. Although OPN can activate the proinflammatory p38 MAPK pathway, leading to neuronal cell death and the release of inflammatory cytokines, it can also support neuronal survival and resilience by activating Akt. Disruption of Akt signaling in pathological conditions can drive neurons toward apoptosis and stimulate inflammatory cytokine production [21].

Moreover, OPN serves as a signaling molecule that enables communication between astrocytes and microglia. CD44, an OPN receptor upregulated on activated astrocytes, is closely linked to elevated OPN levels. Astrocytic activation and subsequent CD44/OPN signaling contribute to the creation of a proinflammatory environment and synaptic dysfunction. IL‐33, an anti‐inflammatory cytokine, demonstrates the potential to counteract the effects of CD44/OPN signaling, suggesting avenues for therapeutic intervention in neuroinflammatory conditions [21].

#### 3.3.5 OPN and Macrophage Chemotaxis

OPN exerts a potent chemoattractant effect on macrophages, contributing to inflammatory responses within the brain. The expression of OPN is closely associated with the infiltration of peripheral myeloid cells into the brain in response to stimuli, highlighting its involvement in immune cell recruitment. The secretion of OPN by microglia in close proximity to Aβ plaques strongly implicates this cytokine in the inflammatory processes and synaptic loss that are characteristic of AD [22]. The secreted isoform of OPN is the predominant pathogenic form in AD, as supported by its localization within the Golgi apparatus. These findings emphasize the critical role of extracellular OPN in the pathogenesis of AD [21].

#### 3.3.6 OPN and ADAM17

ADAM17 functions as a potential ligand for OPN and is implicated in the proteolytic shedding of various membrane‐bound proteins, thereby modulating signaling pathways crucial for inflammation and cell survival [15, 23, 24]. Notably, ADAM17‐mediated cleavage of TREM2 results in the release of soluble TREM2, leading to a decreased presence of TREM2 on the microglial surface [25]. This process hinders microglial responses and impairs Aβ phagocytosis by disrupting TREM2 signaling. Additionally, ADAM17 is involved in the cleavage of the membrane‐bound precursor of TNF‐α (pro‐TNF‐α) to generate its bioactive soluble form (sTNF‐α), playing a pivotal role in regulating TNF‐α–mediated inflammatory processes [26].

The proposed interplay between ADAM17 and OPN is suggested to be essential in driving inflammation and modulating microglial activity. Consequently, the SPP1 deficiency alleviates Aβ accumulation and attenuates neuroinflammation as a potential outcome of this interaction [15].

#### 3.3.7 OPN and Microglial Activation

##### 3.3.7.1 OPN Suppresses TREM2/TAM Lysosomal Phagocytic Pathway

In a comprehensive study, the findings indicated that the absence of OPN resulted in anti‐inflammatory responses within microglia, leading to a reduction in Aβ, decreased neurodystrophy, and an improvement in cognitive function in humans. Notably, in CD11c + microglia, heightened OPN/αvβ3 signaling was associated with the inhibition of the TREM2/TAM (Axl and Mer) lysosomal phagocytic pathway, which consequently impaired the ability of these microglia to phagocytose Aβ plaques. This ultimately led to a reduced capacity for Aβ phagocytosis in microglia expressing OPN [17, 27].

Conversely, microglia lacking OPN expression were characterized by diminished proinflammatory gene expression and an increase in phagocytosis‐related genes, resulting in a gene expression profile resembling that of protective disease‐associated microglia (DAM) [28]. Furthermore, it was observed that OPN played a role in enhancing caspase‐1 activity and IL‐1β production, indicating its involvement in inflammasome activation through the αvβ3 integrin receptor.

The cognitive improvements observed in humans, along with the comprehensive data from the study, suggest the pivotal role of OPN in regulating microglial function and its potential as a therapeutic target for neurodegenerative conditions [27].

##### 3.3.7.2 OPN And TREM1

TREM1, a type of pattern recognition receptor predominantly expressed on myeloid cells, plays a pivotal role in amplifying inflammatory responses. TREM1 triggers a series of intracellular signaling cascades, ultimately leading to the activation of downstream effectors. This signaling pathway culminates in the vigorous production of proinflammatory cytokines and chemokines, thereby intensifying the overall inflammatory response. This action of TREM1 substantially contributes to neuronal damage and exacerbates disease progression [29].

Emerging findings indicate a prospective interrelation between TREM1 and OPN in the pathogenesis of AD. Both molecules are expressed in myeloid cell populations that infiltrate the brain through a CCR2‐dependent mechanism. Although this co‐localization implies a potential synergistic impact on neuroinflammation, further research is needed to elucidate the precise mechanisms governing the interaction between TREM1 and OPN [18].

### 3.4 Neuroprotective

#### 3.4.1 Overview

We reviewed 15 studies to evaluate the potential neuroprotective role of OPN in AD. Our review revealed that four studies directly supported the protective effect of OPN in AD pathology. Three of these studies utilized both in vivo and in vitro models to assess the impact of OPN. Control groups included wild‐type mice in all four primary studies. Detailed description of studies summarizes in Table 4.

#### 3.4.2 OPN and Macrophage‐Mediated Aβ Clearance

In the intricate process of Aβ clearance, OPN has been identified as a pivotal factor. It has been observed that the phagocytosis of Aβ by infiltrating macrophages is OPN dependent. Additionally, matrix metalloproteinase‐9 (MMP‐9) contributes to this process by cleaving OPN into its C‐terminal, known as its immunomodulatory fragment [30].

Experimental data have demonstrated that the genetic ablation of OPN leads to impairment of macrophage‐mediated Aβ phagocytosis, and this deficit can be ameliorated through the exogenous administration of recombinant osteopontin (rOPN). Furthermore, the activation of macrophages and microglia for Aβ clearance has been linked to the TREM2/SYK signaling pathway, which may be activated by OPN [12]. It is reasonable to hypothesize that the influence of MMP‐9 on the cleavage of OPN to its C‐terminal fragment modulates OPN's role in promoting Aβ phagocytosis [30].

Moreover, the study suggests a correlation between elevated levels of OPN and reduced expression of ROS, specifically iNOS and NO, indicating an anti‐inflammatory effect within brain tissue. These data suggest that OPN plays a role in the anti‐inflammatory process and contributes to ameliorating Aβ accumulation [30].

#### 3.4.3 OPN and Synaptogenesis

Expanding upon the previous findings on OPN's role in Aβ clearance, subsequent research focused on OPN's involvement in synaptic maintenance. By quantifying presynaptic (VGluT1) and postsynaptic (PSD95) markers, the study demonstrated a correlation between OPN and synaptic integrity [31].

Specifically, in environments exposed to Aβ oligomers, OPN and MMP‐9 levels were strongly associated with synaptic density and cognitive function, suggesting a potential role for macrophage‐derived OPN in synaptogenesis. However, it is plausible that the C‐terminal fragment of OPN, generated through MMP‐9‐mediated cleavage, may be the primary contributor to this synaptic protective effect [30].

#### 3.4.4 OPN and Aβ Vascular Clearance

Increased OPN levels in specific conditions have been linked to the promotion of angiogenesis, leading to enhanced clearance of vascular Aβ. Quen et al. conducted an investigation into the influence of MyD88 deletion on OPN expression and cerebrovascular function. MyD88 serves as a crucial regulator of innate immunity, activating toll‐like receptor (TLR) signaling pathways [32], thereby inducing the production of proinflammatory cytokines in response to various stimuli, including pathogens and Aβ [33].

Deletion of MyD88 resulted in heightened expression of angiogenic genes, including OPN and insulin–like growth factor‐1 (IGF‐1). Concurrently, expansion of cerebrovascular tissue was observed, facilitating an increase in the clearance of vascular Aβ. Furthermore, MyD88 deficiency upregulated the expression of low–density lipoprotein receptor‐related protein 1 (LRP1) in brain capillary pericytes. LRP1 plays a critical role in the efflux of Aβ from the brain into the vasculature, thereby contributing to the clearance of Aβ [33].

These findings collectively imply that MyD88 deletion mitigates Alzheimer's pathology through a dual mechanism: (1) suppression of inflammatory gene expression (TNF‐α, IL‐1β) and (2) promotion of cerebrovascular health via increased angiogenesis (OPN, IGF‐1) and enhanced Aβ clearance (LRP1).

### 3.5 RoB Assessment

The methodological quality of the included studies was assessed using the SYRCLE RoB tool, which evaluates 10 items across domains such as selection, performance, detection, attrition, and reporting biases. A detailed summary of the findings is provided in Data S2.

## 4 Discussion

**FIGURE 2:**
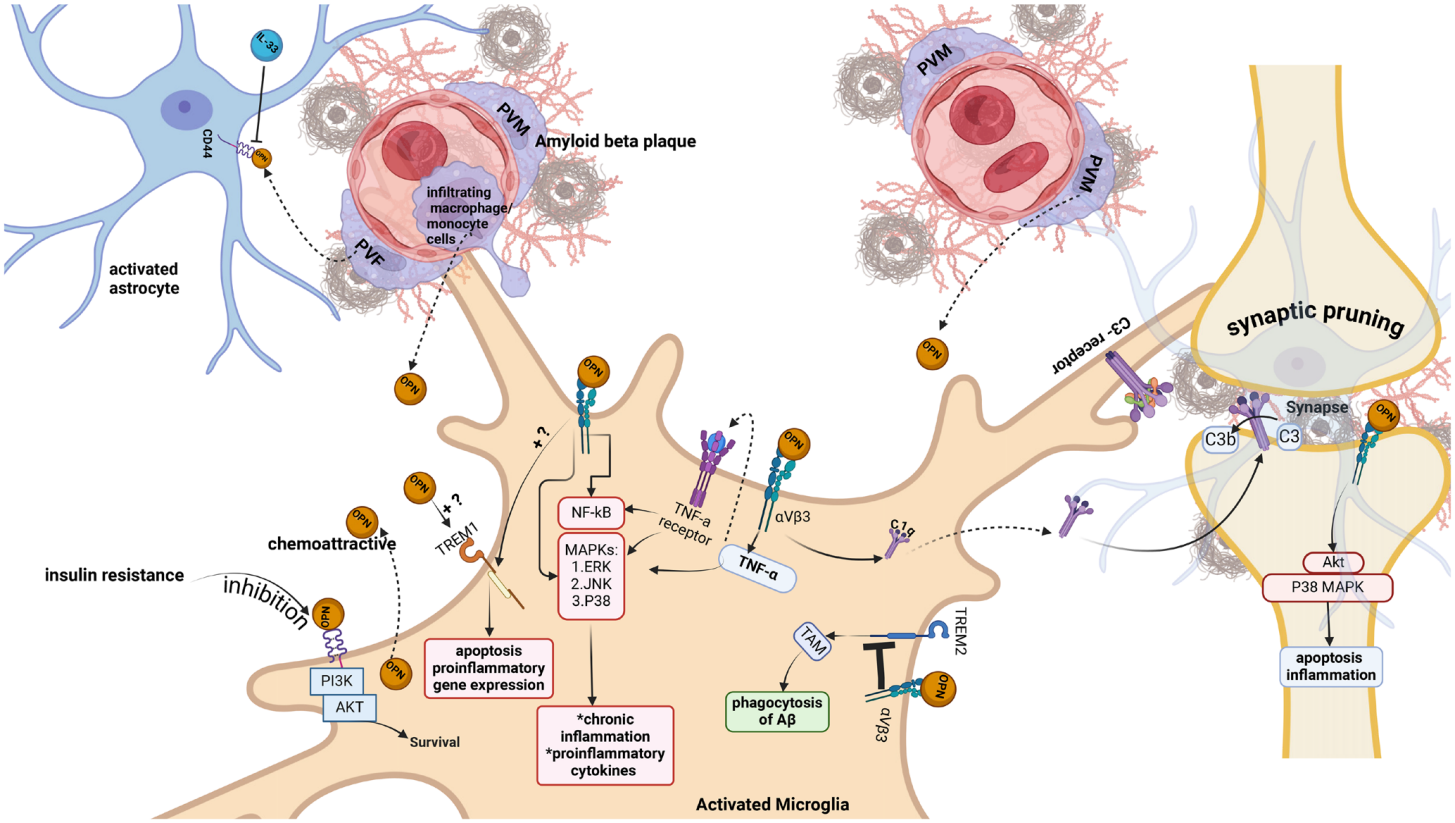
Neuroinflammation role of OPN in Alzheimer's disease pathology. This figure illustrates how OPN contributes to AD pathology by promoting inflammatory responses, synaptic pruning, and activating intracellular signaling pathways that lead to neuronal apoptosis. In pathological conditions such as AD, where amyloid plaques are present, microglia and PVM/PVF cells secrete OPN. As a chemoattractive cytokine, OPN recruits more activated microglia and infiltrating myeloid cells (macrophages/monocytes), which in turn secrete more OPN, exacerbating the inflammatory cascade. OPN exerts its effects through its receptors, integrin, and activating various pathways, including the complement cascade, TNF‐α, NF‐κB, and MAPK signaling pathways, all of which contribute to inflammatory responses and cell death. Additionally, by inhibiting TREM2/TAM signaling, OPN impairs the phagocytosis of amyloid‐beta. In neurons, OPN can also activate the Akt and P38 MAPK pathways, leading to apoptosis and inflammation. Also, it shows the microglia and astrocyte interaction to synaptic pruning by molecular cross talk. OPN and CD44 promote the inflammatory state of astrocytes, leading to the secretion of C1q, a key initiator of the complement cascade, which mediates synaptic loss. Created with BioRender.com.

**FIGURE 3:**
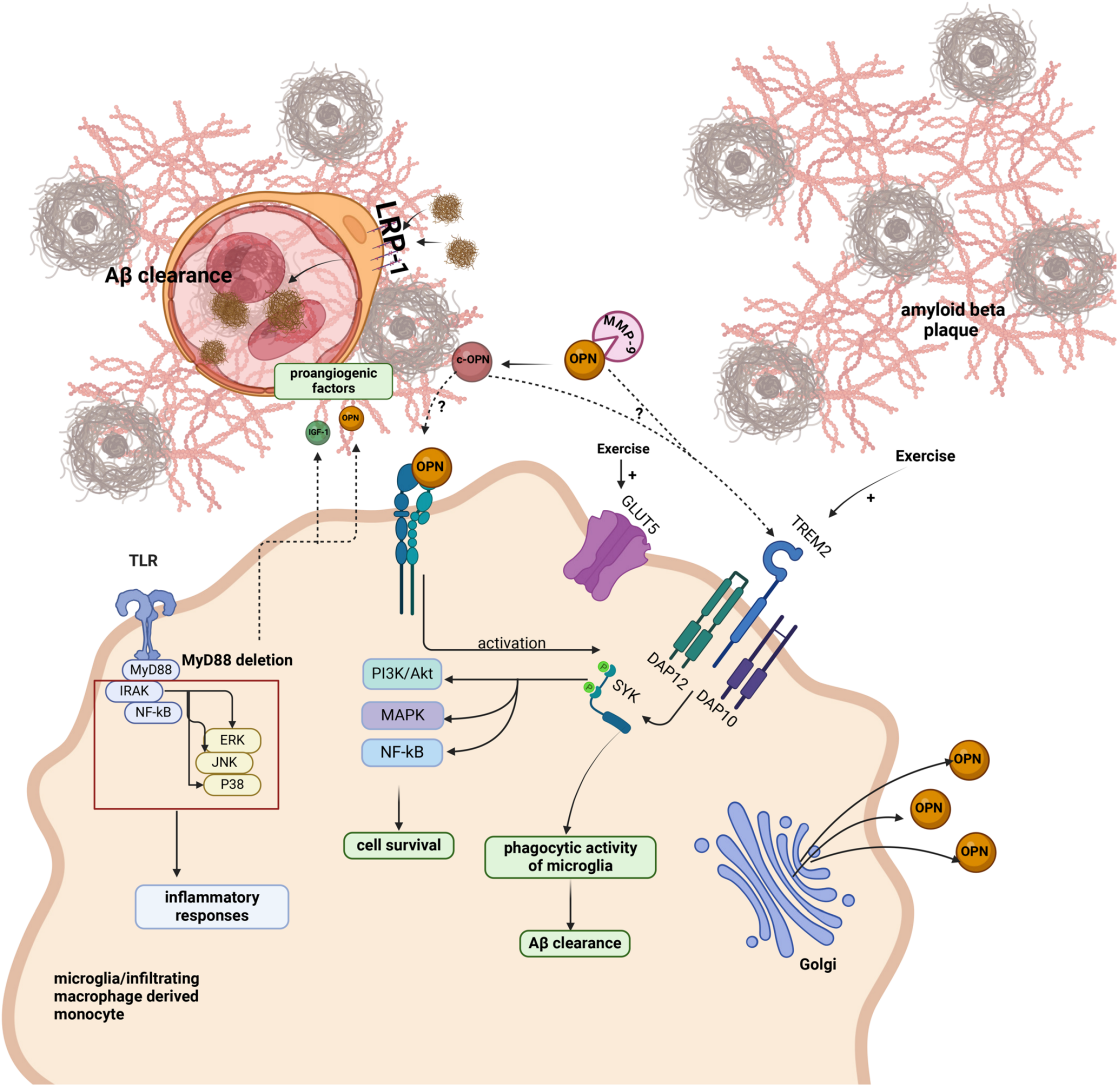
Neuroprotective role of OPN in Alzheimer's disease pathology. This figure illustrates the neuroprotective mechanisms of OPN, including the enhancement of amyloid‐beta clearance through phagocytosis mediated by the TREM2/SYK signaling pathway in microglia and infiltrating macrophage‐derived monocytes. Additionally, OPN increases cerebral vascularization as a proangiogenic factor. We propose that c‐OPN, degraded by MMP‐9, exerts protective effects. Created with BioRender.com.

Our review brings to light the paradoxical function of OPN in AD. The existing literature predominantly emphasizes OPN's potential contribution to neurodegeneration, as illustrated in Figure 2. However, a subset of studies posits its capacity for neuroprotection, as depicted in Figure 3. OPN may play a role in either promoting neuronal degeneration or providing neuroprotective benefits based on the surrounding milieu [35]. Notably, research by Rentsendorj et al. elucidated that the process of Aβ phagocytosis by macrophages is OPN dependent, facilitated through the activation of the TREM2/SYK signaling axis [30]. We posit that this protective role of OPN is mediated by OPN‐c, an immunomodulatory fragment generated by MMP‐9, which initiates Aβ phagocytosis [7].

OPN fragments generated through cleavage by proteolytic enzymes, namely, thrombin and matrix metalloproteinases (MMPs), exhibit unique biological properties. Thrombin‐induced cleavage yields OPN‐N, granting implications in neuroinflammation, whereas MMP‐9‐mediated cleavage results in the formation of OPN‐c, leading to immunomodulatory effects and interaction with CD44 [36].

A study conducted by Quan et al. revealed that the deletion of MyD88 led to heightened expression of OPN and IGF‐1 [33]. These findings suggest that mitigating inflammation through inhibition of MyD88 could potentially alter the functional behavior of OPN, transforming it from a neurodegenerative factor to a neuroprotective agent. Moreover, the concurrent presence of OPN and IGF‐1, both possessing proangiogenic properties, may influence the overall impact of OPN on diseased states [37].

In a separate study, Zhang et al. noted elevated levels of OPN in the AD group with physical activity (AD‐Run), where it seemed to exhibit a protective role. We hypothesize that physical exercise could potentially reduce inflammation, thus reshaping the milieu in a manner that steers OPN toward a neuroprotective function [12].

The signaling pathways triggered by OPN exhibit receptor–specific activation effects. Binding of OPN to integrin receptors leads to the activation of ERK, P38 MAPK, and JNK signaling cascades, resulting in the generation of inflammatory cytokines and induction of apoptosis, both of which are neurotoxic processes [6]. Conversely, interaction of OPN with the CD44 receptor activates the PI3K/Akt signaling pathway, fostering neuronal survival and demonstrating neuroprotective properties [38]. However, this protective mechanism may be compromised in the presence of insulin resistance, a condition in individuals with metabolic disorders [39] and neurodegenerative conditions [6].

The neuroprotective functions of microglia in Aβ phagocytosis may be compromised in conditions characterized by inflammation and Aβ oligomer accumulation, resulting in notable impacts on synaptic activity. One key mechanism through which OPN contributes to synaptic loss involves the initiation of the complement cascade, which leads to the engulfment of synapses. Specifically, the secreted form of OPN (sOPN) plays a pivotal role in triggering this cascade, thereby prompting the recognition and subsequent elimination of synapses by microglia. Furthermore, activated astrocytes release C1q, suggesting a potential implication of OPN in astrocyte–mediated synaptic elimination [40].

Recent research investigating the involvement of astrocytes and microglia in synaptic pruning within AD mouse models has revealed distinct patterns of synaptic puncta distribution between these two cell types, indicative of a functional ‘division of labor’ in synaptic pruning tasks. Notably, astrocytes appear to target the removal of excitatory synapses predominantly, whereas microglia exhibit a greater role in clearing inhibitory synapses. This differential involvement underscores the intricate interplay between these glial cells and their specific contributions to sustaining synaptic integrity and adaptability in the context of neurodegenerative disorders such as AD. The study underscores the significance of both astrocytes and microglia in the process of synaptic pruning, with each cell type demonstrating a preference for different synaptic subtypes [41].

Inflammatory conditions result in the upregulation of CD44 expression on astrocytes, which enhances their interaction with OPN released by activated microglia. This molecular communication between microglia and astrocytes drives astrocytes toward an inflammatory state, exacerbating synaptic loss. In this activated state, astrocytes lose their ability to support synaptic structures and instead contribute to the complement‐driven phagocytosis of synapses by microglia [40]. Remarkably, research indicates that the receptor TREM2 may mitigate synaptic loss by binding to C1q, thus preventing excessive complement activation and subsequent synaptic elimination [42].

The interplay between synaptic dysfunction and neuroinflammation establishes a positive feedback loop, where each factor reinforces the other, ultimately contributing to the development of AD pathology [43].

Microglial activation can be modulated by OPN through its regulatory effects on TREM receptors. OPN has been shown to inhibit the TREM2/Tyrobp‐associated membrane protein (TAM) signaling pathway, which plays a crucial role in the phagocytosis of Aβ plaques [17]. Simultaneously, OPN can enhance proinflammatory responses by likely activating TREM1 [18]. This interaction between OPN and TREM1 is believed to shift microglial activation toward a more proinflammatory and potentially neurotoxic phenotype. Consequently, this dysregulated activation may impede the ability of microglia to effectively clear Aβ, leading to its accumulation and exacerbating the progression of AD pathology [44].

Moreover, OPN is known to induce microglial release of the proinflammatory cytokine TNF‐α [17], leading to a series of inflammatory reactions mediated by the NF‐κB and MAPK signaling pathways [26]. The activation of these pathways ultimately leads to the synthesis of proinflammatory cytokines such as interleukin‐1 (IL‐1), interleukin‐6 (IL‐6), and interleukin‐8 (IL‐8), resulting in chronic inflammation and subsequent neuronal apoptosis. Additionally, chronic inflammation and cytokine production may also be initiated by triggering TREM1 activation [26].

Given the known regulation of TNF‐α expression by the p38 and NF‐κB signaling pathways [26], it is theorized that OPN plays a role in activating these signaling cascades [8]. OPN serves as a biomarker of pathogenic microglia, differentiating these cells from their neuroprotective counterparts [17]. The upregulation of integrin alpha X (ITGAX) and secreted phosphoprotein 1 (SPP1) in activated microglia highlights the importance of OPN–induced receptor interactions in microglial activation [45].

TGF‐β, produced by astrocytes and neurons, plays a crucial role in modulating inflammatory responses in microglial cells. It suppresses the production of cytokines and ROS by microglia, thereby promoting phagocytosis and enhancing neuroprotection. Nevertheless, prolonged activation of microglia can reduce their sensitivity to TGF‐β, leading to heightened cytotoxicity and impaired clearance of Aβ. Consequently, although microglia demonstrate protective actions during the initial phases of disease progression, sustained activation drives them towards a harmful phenotype in chronic contexts, characterized by a diminished capacity for functional adaptability [46].

Although OPN has been suggested to play a neuroprotective role, clinical trials and in vitro studies utilizing rOPN for AD treatment have produced inconclusive outcomes, as significant improvements compared to control groups were not observed [21]. The bulk of evidence points toward OPN being involved in neurodegenerative processes, yet its potential protective functions should not be ignored. The multifaceted nature of OPN, presenting both detrimental and beneficial effects, is influenced by factors such as the source cell, target cell, and specific receptor interactions. Notably, under nonpathological conditions, OPN can enhance microglial phagocytosis, highlighting the intricate and context‐specific roles of this molecule in AD [9, 35]. Although the diagnostic potential of OPN is still being investigated, recent studies propose the utilization of cerebrospinal fluid (CSF) OPN in presymptomatic AD and mild cognitive impairment [47].

Our exhaustive literature review indicates that the dual functionality of OPN is reliant on various factors including the disease stage, environmental context (chronic or acute inflammation), OPN–secreting cell types, and the receptors OPN interacts with. The impact of OPN is influenced by the inflammatory or protective nature of the conditions, leading to a synergistic effect.

### 4.1 Future Direction

Recent studies highlight the diagnostic potential of CSF OPN in mild cognitive impairment. For instance, in the Presymptomatic Evaluation of Experimental or Novel Treatments for Alzheimer's Disease cohort, Quesnel et al. demonstrated that elevated CSF OPN levels can serve as early indicators of synaptic dysfunction, tau deposition, and neuronal loss in cognitively unimpaired elderly individuals with a parental history of AD. These findings underscore the relevance of OPN as a potential biomarker for early AD detection [48].

Although the therapeutic application of OPN in AD remains underexplored, one study included in our review utilized recombinant OPN but did not yield significant findings [21]. This outcome suggests a need for more nuanced research into therapeutic targets, with careful attention to timing and context. Emerging evidence and expert opinions emphasize the potential of targeting OPN in the synaptic pruning pathway [17]. Although OPN has not been directly established as a therapeutic target in AD, its inhibition has demonstrated promising outcomes in other conditions, such as breast and colorectal cancers, where it has led to tumor suppression [49, 50]. Notably, intranasal administration of OPN has shown to influence microglial polarization and enhance phagocytic activity via RGD‐dependent mechanisms, further reinforcing its potential as a therapeutic candidate [51]. These findings open new avenues for investigating OPN in the context of neurodegenerative diseases and beyond.

### 4.2 Limitations

This study provides important insights into the role of OPN in AD, but several limitations should be noted. Most of the included studies are preclinical, relying on in vivo and in vitro models that do not fully replicate the complexity of AD in humans, highlighting the need for validation in longitudinal human studies. The studies exhibited significant heterogeneity in experimental designs, including differences in animal models, cell lines, methods of assessing OPN levels, and outcome measures. This variability limited the ability to draw consistent conclusions and precluded quantitative synthesis, such as a meta‐analysis. Furthermore, the lack of standardized effect measures, such as mean differences or risk ratios, complicated result aggregation and comparison. There is also the potential for publication bias, which may have led to an overrepresentation of findings supporting OPN's role in AD while underreporting negative or inconclusive results.

Many studies focused on single disease stages, restricting the understanding of OPN's dual role as both neuroprotective and neuroinflammatory across AD progression. Some studies also lacked detailed reporting on methodological aspects, such as randomization, allocation concealment, and blinding, which could introduce bias and affect reliability. Additionally, although mechanisms of OPN were explored, limited attention was given to its direct therapeutic applications or how targeting OPN signaling could be translated into clinical strategies for AD treatment. Addressing these limitations in future research through more robust, standardized, and human‐centered approaches is crucial to advancing our understanding of OPN's role in AD and its potential as a therapeutic target.

## 5 Conclusion

OPN represents a paradoxical molecule in AD, capable of both protecting neurons and contributing to their degeneration. Future research should focus on identifying the conditions under which OPN's protective mechanisms can be enhanced while mitigating its degenerative effects. Targeting specific OPN signaling pathways may offer novel therapeutic strategies for slowing AD progression and preserving cognitive function. The complexity of OPN's role in AD stems from its involvement in multiple signaling pathways, inflammatory responses, and interactions with various cell types within the brain. Understanding the dual nature of OPN's effects is crucial for developing targeted therapies aimed at modulating its activity.

## Author Contributions

**Zahra Azizan:** writing – original draft, methodology, visualization, data curation, screening, selection, data extraction, advanced search. **Maryam Bazrgar:** supervision, project administration, methodology, conceptualization, selection, data extraction, validation. **Mohammad Hossein Harirchian:** validation, conceptualization. **Narges Bazgir:** screening, selection, data extraction, advanced search. **Sadra Habibi Moini:** writing – review and editing. **Sara Ghaseminejad‐Kermani:** visualization, data curation, review and editing. **Kamran Safa:** visualization, data curation, review and editing. **Azam Eshaghian‐dorcheh:** methodology. All authors reviewed the manuscript.

## Consent

The authors have nothing to report.

## Conflicts of Interest

The authors declare no conflicts of interest.

## Institutional Review Board Statement

The authors have nothing to report.

## Supporting information


Data S1.

Data S2.

## Data Availability

The data that support the findings of this study are available from the corresponding author.
