# Supplementary material for: Osteopontin in Alzheimer's Disease: A Double‐Edged Sword in Neurodegeneration and Neuroprotection—A Systematic Review
Source: CNS Neurosci Ther. 2025 Feb 17;31(2):e70269. doi: 10.1111/cns.70269 (PMC11831194; doi:10.1111/cns.70269)
Supplement: Supplementary file 1 — Data S1. [file CNS-31-e70269-s001.docx]

**Supplementary 1. Search strategy**

**Search date: 1 march 2024🡪update search 1 July 2024**

**Pubmed:171 🡪 176**

("Alzheimer Disease"[MeSH Terms] OR "Alzheimer* Disease*"[Text Word] OR "Alzheimer Sclerosis"[Text Word] OR "Acute Confusional Senile Dementia"[Text Word] OR "Presenile Dementia"[Text Word] OR "Alzheimer Dementia"[Text Word] OR "Alzheimer*"[Text Word] OR "dementia*"[Text Word] OR "Senile Dementia"[Text Word] OR "Alzheimer Syndrome"[Text Word] OR "Presenile Dementia"[Text Word] OR "AD"[Text Word]) AND ("Osteopontin"[MeSH Terms] OR "Osteopontin"[Text Word] OR "Bone Sialoprotein I"[Text Word] OR "Sialoprotein I"[Text Word] OR "Sialoprotein 1"[Text Word] OR "Bone Sialoprotein 1"[Text Word] OR "Secreted Phosphoprotein 1"[Text Word] OR "Uropontin"[Text Word] OR "SPP1"[Text Word] OR "OPN"[Text Word])

**Scopus:225 🡪 237( 3 conference paper)**

(TITLE-ABS-KEY ( osteopontin ) OR TITLE-ABS-KEY ( opn ) OR TITLE-ABS-KEY ( bone AND sialoprotein AND i ) OR TITLE-ABS-KEY ( sialoprotein AND i ) OR TITLE-ABS-KEY ( sialoprotein 1 ) OR TITLE-ABS-KEY ( bone AND sialoprotein 1 ) OR TITLE-ABS-KEY ( secreted AND phosphoprotein 1 ) OR TITLE-ABS-KEY ( uropontin ) OR TITLE-ABS-KEY ( spp1 ) ) AND (TITLE-ABS-KEY ( alzheimer&apos;s AND disease ) OR TITLE-ABS-KEY ( alzheimer ) OR TITLE-ABS-KEY ( alzheimer&apos;s ) OR TITLE-ABS-KEY ( alzheimer AND sclerosis ) OR TITLE-ABS-KEY ( acute AND confusional AND senile AND dementia ) OR TITLE-ABS-KEY ( alzheimer AND disease ) OR TITLE-ABS-KEY ( presenile AND dementia ) OR TITLE-ABS-KEY ( alzheimer AND dementia ) OR TITLE-ABS-KEY ( dementia ) OR TITLE-ABS-KEY ( senile AND dementia ) OR TITLE-ABS-KEY ( alzheimer AND syndrome ) OR TITLE-ABS-KEY ( presenile AND dementia ))

**Embase:388 🡪 406**

('Alzheimer disease'/exp OR 'alzheimer* disease*':ti,ab,kw,de,dn,df,mn,tn OR 'alzheimer sclerosis':ti,ab,kw,de,dn,df,mn,tn OR 'acute confusional senile dementia':ti,ab,kw,de,dn,df,mn,tn OR 'presenile dementia':ti,ab,kw,de,dn,df,mn,tn OR 'alzheimer dementia':ti,ab,kw,de,dn,df,mn,tn OR 'alzheimer*':ti,ab,kw,de,dn,df,mn,tn OR 'dementia*':ti,ab,kw,de,dn,df,mn,tn OR 'senile dementia':ti,ab,kw,de,dn,df,mn,tn OR 'alzheimer syndrome':ti,ab,kw,de,dn,df,mn,tn OR 'presenile dementia':ti,ab,kw,de,dn,df,mn,tn OR 'ad':ti,ab,kw,de,dn,df,mn,tn) AND ('osteopontin'/exp OR 'osteopontin':ti,ab,kw,de,dn,df,mn,tn OR 'bone sialoprotein i':ti,ab,kw,de,dn,df,mn,tn OR 'sialoprotein i':ti,ab,kw,de,dn,df,mn,tn OR 'sialoprotein 1':ti,ab,kw,de,dn,df,mn,tn OR 'bone sialoprotein 1':ti,ab,kw,de,dn,df,mn,tn OR 'secreted phosphoprotein 1':ti,ab,kw,de,dn,df,mn,tn OR 'uropontin':ti,ab,kw,de,dn,df,mn,tn OR 'spp1':ti,ab,kw,de,dn,df,mn,tn OR 'opn':ti,ab,kw,de,dn,df,mn,tn)

**+ theses : 1**

**Total : 820**

**Duplication: automatic 370 , manual 17**

**Total Duplications: 387**

**Studies = 436**
