# Supplementary material for: Osteopontin in Alzheimer's Disease: A Double‐Edged Sword in Neurodegeneration and Neuroprotection—A Systematic Review
Source: CNS Neurosci Ther. 2025 Feb 17;31(2):e70269. doi: 10.1111/cns.70269 (PMC11831194; doi:10.1111/cns.70269)
Supplement: Supplementary file 2 — Data S2. [file CNS-31-e70269-s002.docx]

| Table S2. SYRCLE Risk of Bias (RoB) tool assessment of included studies | | | | | | | | | | | |
| --- | --- | --- | --- | --- | --- | --- | --- | --- | --- | --- | --- |
| **No.** | **Study** | **Item 1** | **Item 2** | **Item 3** | **Item 4** | **Item 5** | **Item 6** | **Item 7** | **Item 8** | **Item 9** | **Item 10** |
| **1** | Rentsendorj et al [31] | Unclear | Yes | Yes | Unclear | Yes | Unclear | Yes | Yes | Yes | Yes |
| **2** | Li et al [32] | Unclear | Yes | unclear | Unclear | Yes | Yes | Yes | Yes | Yes | Yes |
| **3** | Gharpure et al [28] | Unclear | Unclear | Unclear | Unclear | Unclear | No | No | Yes | Yes | Yes |
| **4** | Wang et al [27] | Unclear | Unclear | Unclear | Unclear | Unclear | No | Yes | Yes | Yes | Yes |
| **5** | Lai et al [13] | Yes | Yes | Yes | Yes | Unclear | Unclear | Unclear | Yes | Yes | Yes |
| **6** | Qiu et al [17] | Yes | Yes | Unclear | Yes | Yes | No | Yes | Yes | Yes | Yes |
| **7** | Quan et al [34] | Yes | Yes | Unclear | Unclear | Unclear | Unclear | Unclear | Yes | Yes | Yes |
| **8** | Wirths et al [22] | Unclear | Yes | Unclear | Unclear | Unclear | Unclear | Unclear | Yes | Yes | Yes |
| **9** | Zhang et al [12] | Yes | Yes | Yes | Yes | Yes | Yes | Yes | Yes | Yes | Yes |
| **10** | De Schepper et al [15] | Unclear | Yes | Unclear | Unclear | Yes | Unclear | Yes | Yes | Yes | Yes |
| **11** | Hong et al [30] | Unclear | Yes | Unclear | Unclear | Yes | Unclear | Yes | Yes | Yes | Yes |
| **12** | Chuang et al [21] | Unclear | Yes | Unclear | Unclear | Unclear | Unclear | Unclear | Yes | Yes | Yes |
| **13** | Sala Frigerio et al [11] | Unclear | Yes | Unclear | Unclear | Yes | Unclear | Yes | Yes | Yes | Yes |
| **14** | Yang et al [18] | Unclear | Yes | Unclear | Unclear | Unclear | Unclear | Yes | Yes | Yes | Yes |
| **15** | J-H Park et al [10] | Unclear | Unclear | Unclear | Unclear | Unclear | Unclear | Unclear | Yes | Yes | Yes |
| "Yes" indicating "low risk" and "No" indicating "high risk."  Item 1: Was the allocation sequence adequately generated and applied?  Item 2: Were the groups similar at baseline or were they adjusted for confounders in the analysis?  Item3: Was the allocation to the different groups adequately concealed during?  Item 4: Were the animals randomly housed during the experiment?  Item 5: Were the caregivers and/or investigators blinded from knowledge which intervention each animal received during the experiment?  Item 6: Were animals selected at random for outcome assessment?  Item 7: Was the outcome assessor blinded?  Item 8: Were incomplete outcome data adequately addressed?  Item 9: Are reports of the study free of selective outcome reporting?  Item 10: Was the study apparently free of other problems that could result in high risk of bias? | | | | | | | | | | | |
